# Supplementary material for: Real-time transcriptomic profiling in distinct experimental conditions
Source: eLife. 2026 May 5;13:RP98768. doi: 10.7554/eLife.98768 (PMC13143281; doi:10.7554/eLife.98768)

**Supplementary file 1.** **Step-by-step guideline to NanopoReaTA.** *(For additional information, please refer to Wierczeiko et al. 2023 or visit* [*https://github.com/AnWiercze/NanopoReaTA*](https://github.com/AnWiercze/NanopoReaTA)*).*

1. ***Run docker***

docker run -it -p 8080:8080 -v /:/NanopoReaTA_linux_docker stegiopast/nanoporeata:references

1. ***Open in new window***

<http://0.0.0.0:8080>


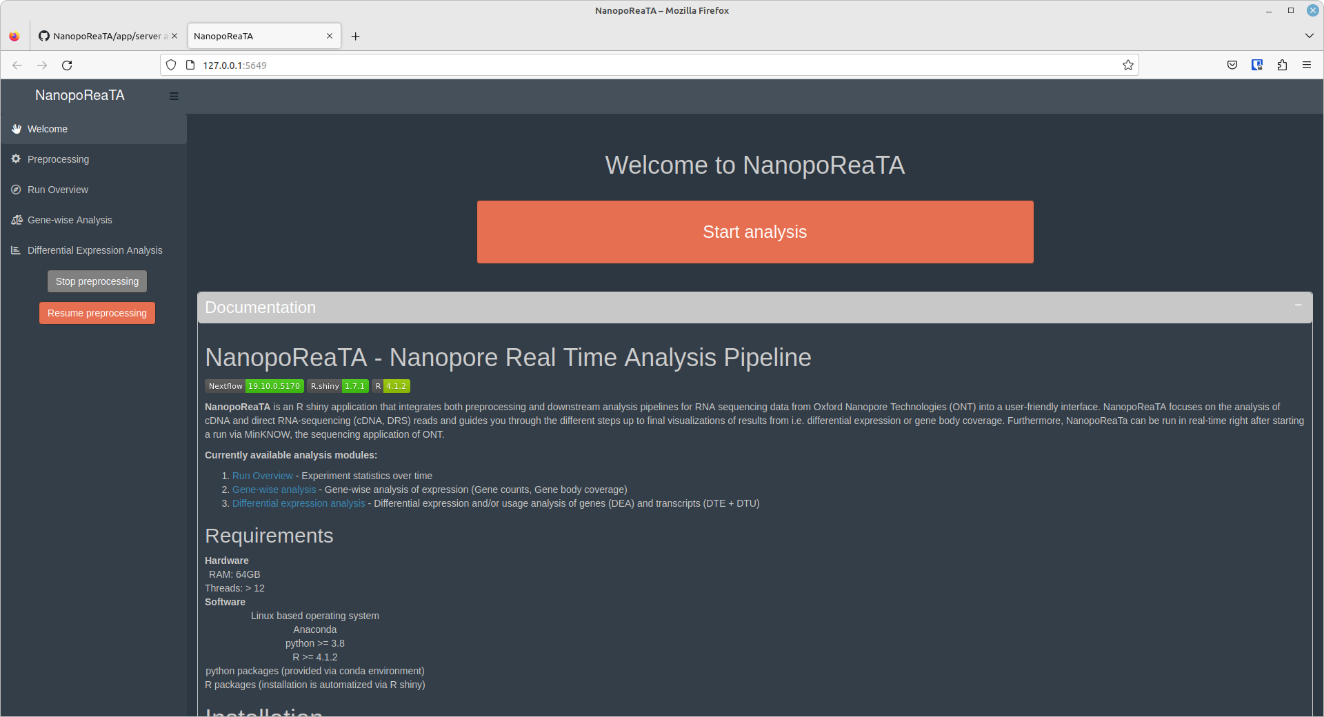


1.
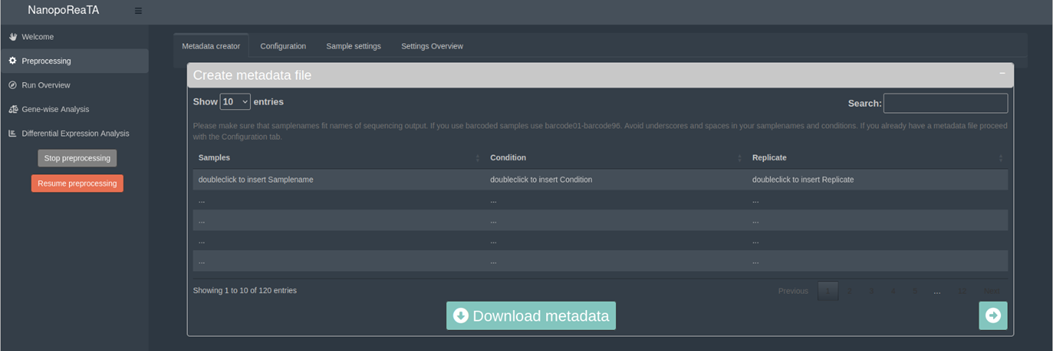
***Metadata creator***
   - insert in Samples the barcodes used with nanopore (e.g. barcode1, barcode2, etc..)
   - insert in Condition the name of tested condition (e.g. Condition A, Condition B)
   (Not recommended to insert “_” in the condition names)
   - insert in Replicate the replicates names (e.g. R1)
   - Download the metadata

   - Users can prepare in advance metadata template from (*example_metadata.txt*):
    <https://github.com/AnWiercze/NanopoReaTA/tree/master/example_conf_files>
2. ***Creation of a configuration file***- Download the configuration template from (*example_config.txt*): <https://github.com/AnWiercze/NanopoReaTA/tree/master/example_conf_files>
   - insert in Condition the name of tested condition (e.g. Condition A, Condition B)
   (No recommended _ in the condition names)
   - insert in Replicate the replicates names (e.g. R1)
   - Download the metadata


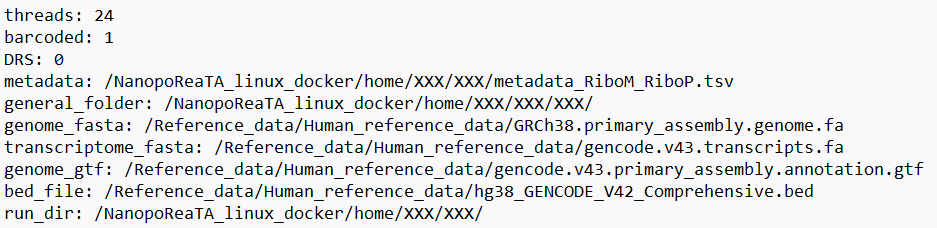


***- Number of threads to use***

**- Barcoded samples. 0 – no; 1 - Yes**

**- Direct RNA sequencing. 0 – no; 1 - Yes**

***- Path to the sequencing folder (MinKnow Output)***

***- Path to the metadata file (.tsv)***

***- *Path to reference trascriptome (.fasta)***

***- *Path to refence genome (.fasta)***

***- *Path to Bed file (.bed)***

***- *Path to Gtf file (.gtf)***

***- Path to NanopoReaTA output folder***

*With the docker image tag "references", all human and mouse reference files needed for NanopoReaTA will be automatically downloaded from GENCODE (~36 GB) and saved in root.

1.
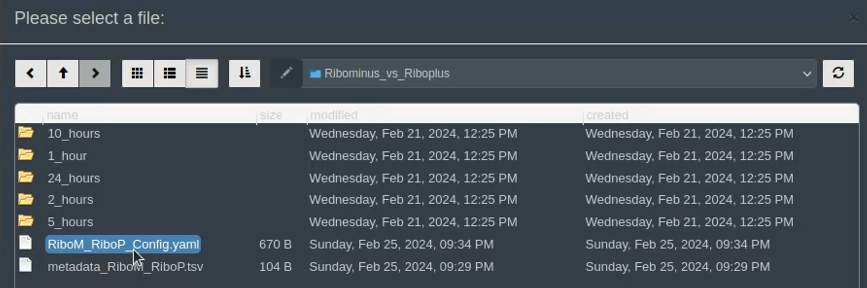

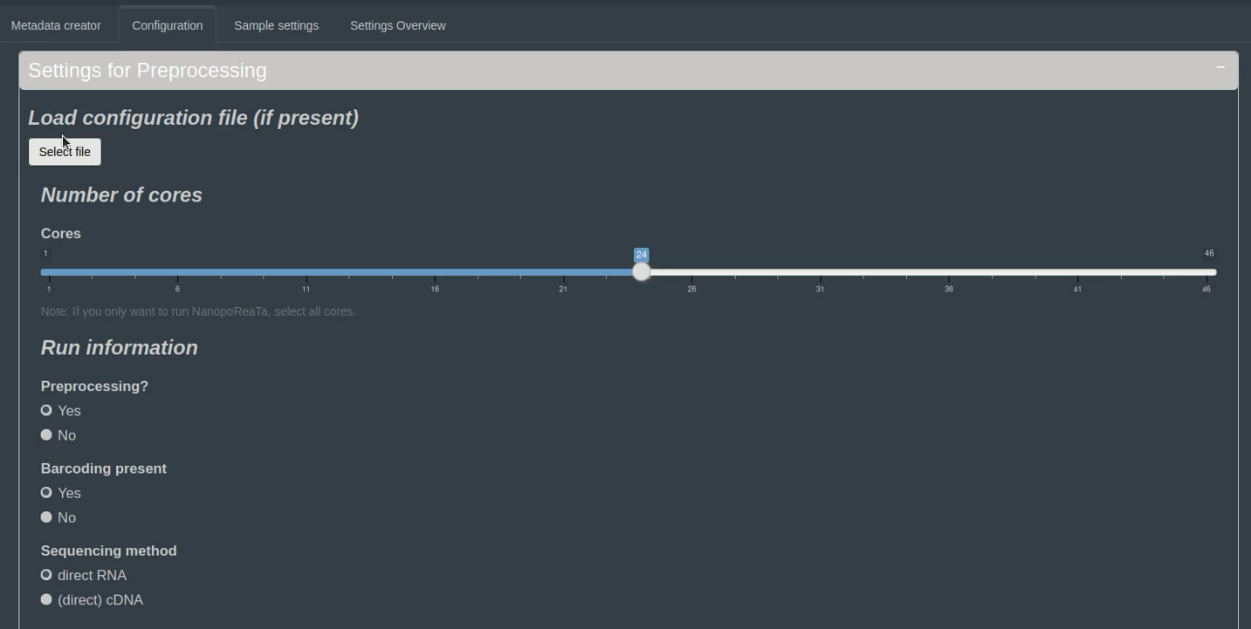
***Loading the configuration file***- open the configuration tab and press on “Select file” to load the configuration file.


   - Select the configuration file (.yaml)


   - Selected files and folder output should upload to the appropriate section
   - Note: if not all of the files are uploaded, you can insert the missing file manually.


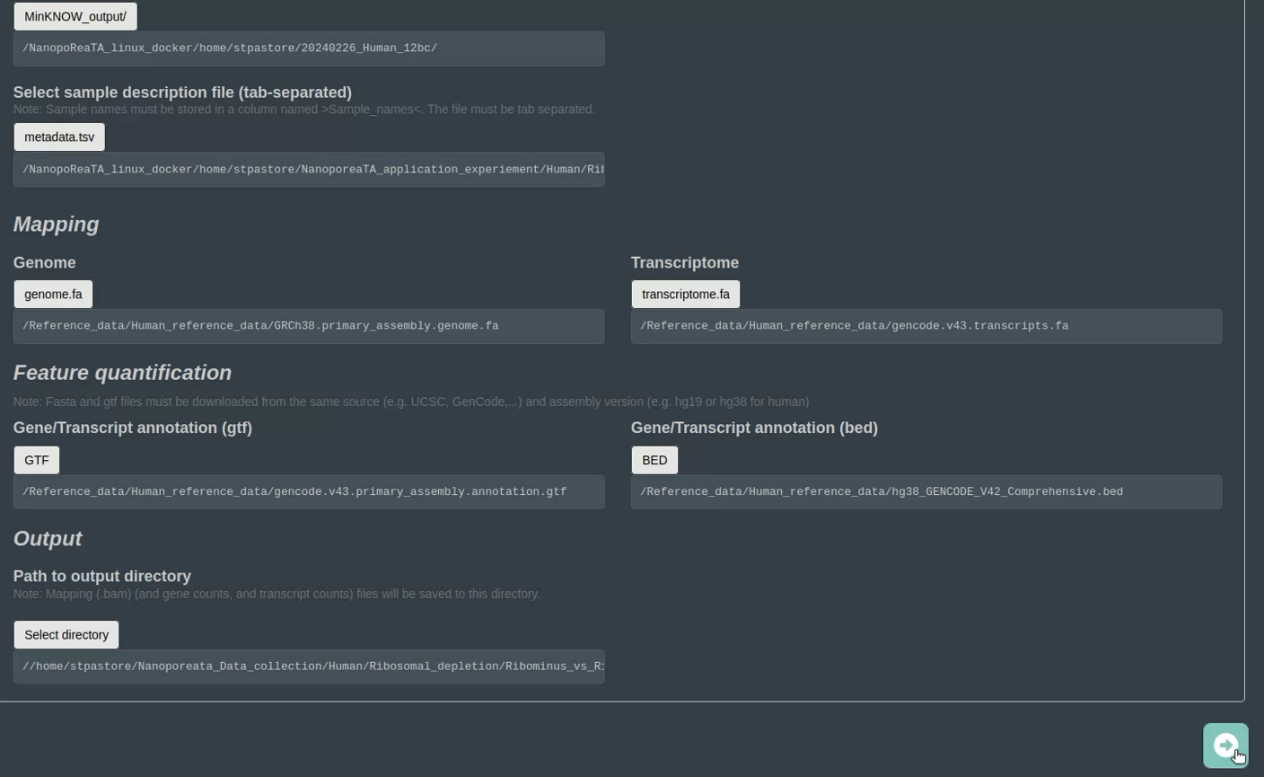


   - Following configuration upload, check in “Sample settings” that all barcodes, conditions and replicates are inserted properly
   - Users can select colors for the specific condition which will be shown in the visualized data
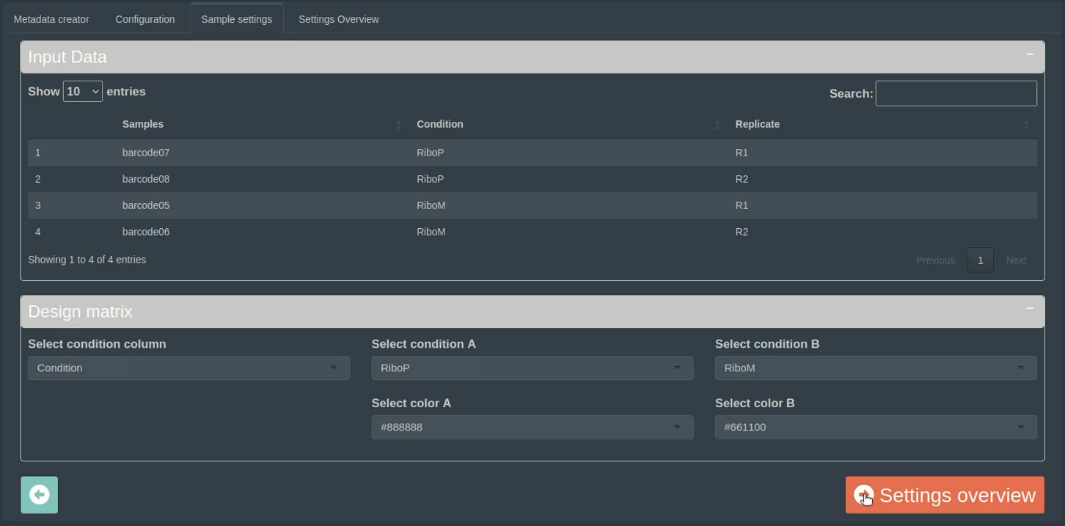
analysis


   - In “Settings Overview”, check that all the details are inserted properly and press “Start” real-time transcriptomic analysis


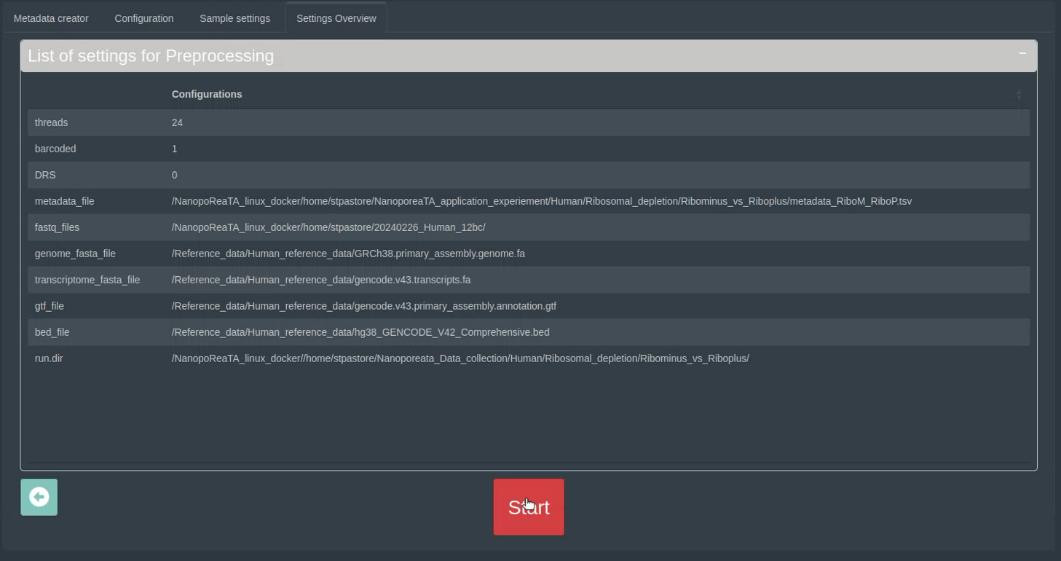

2.
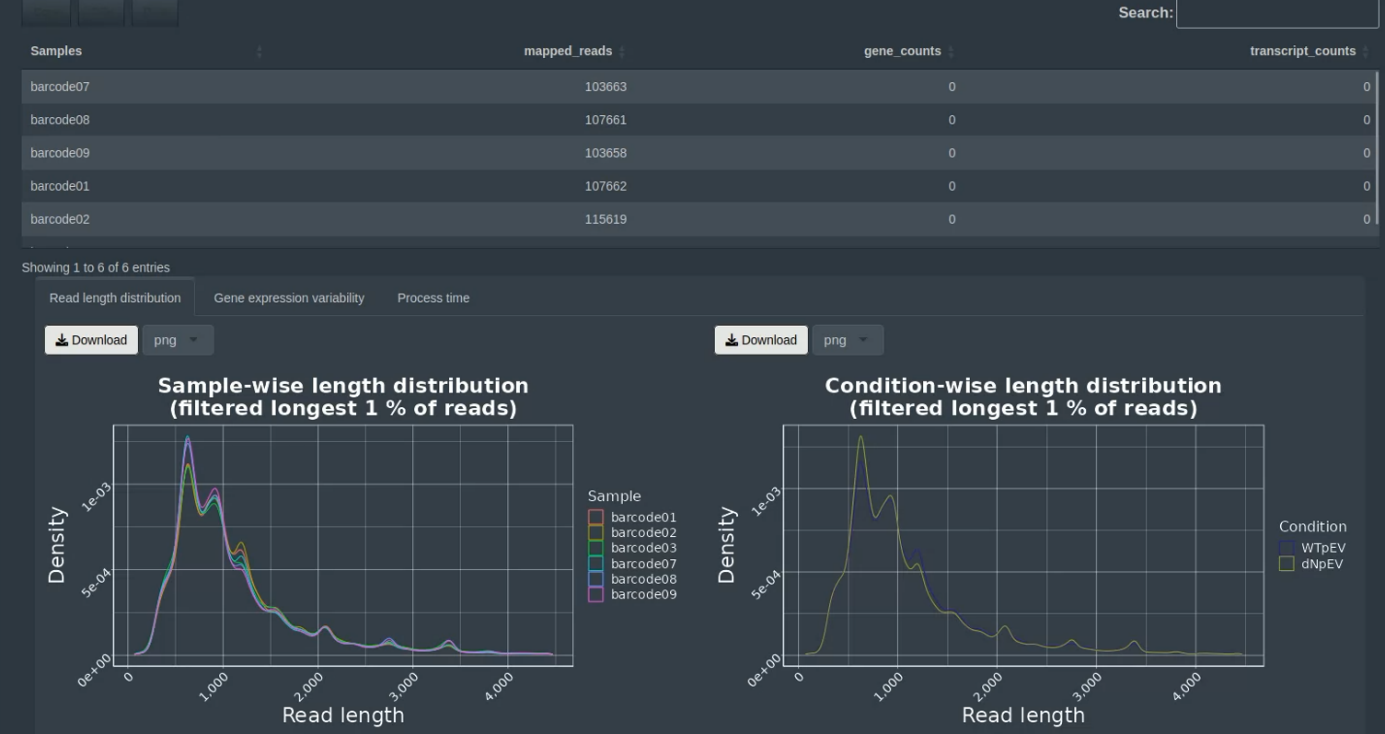

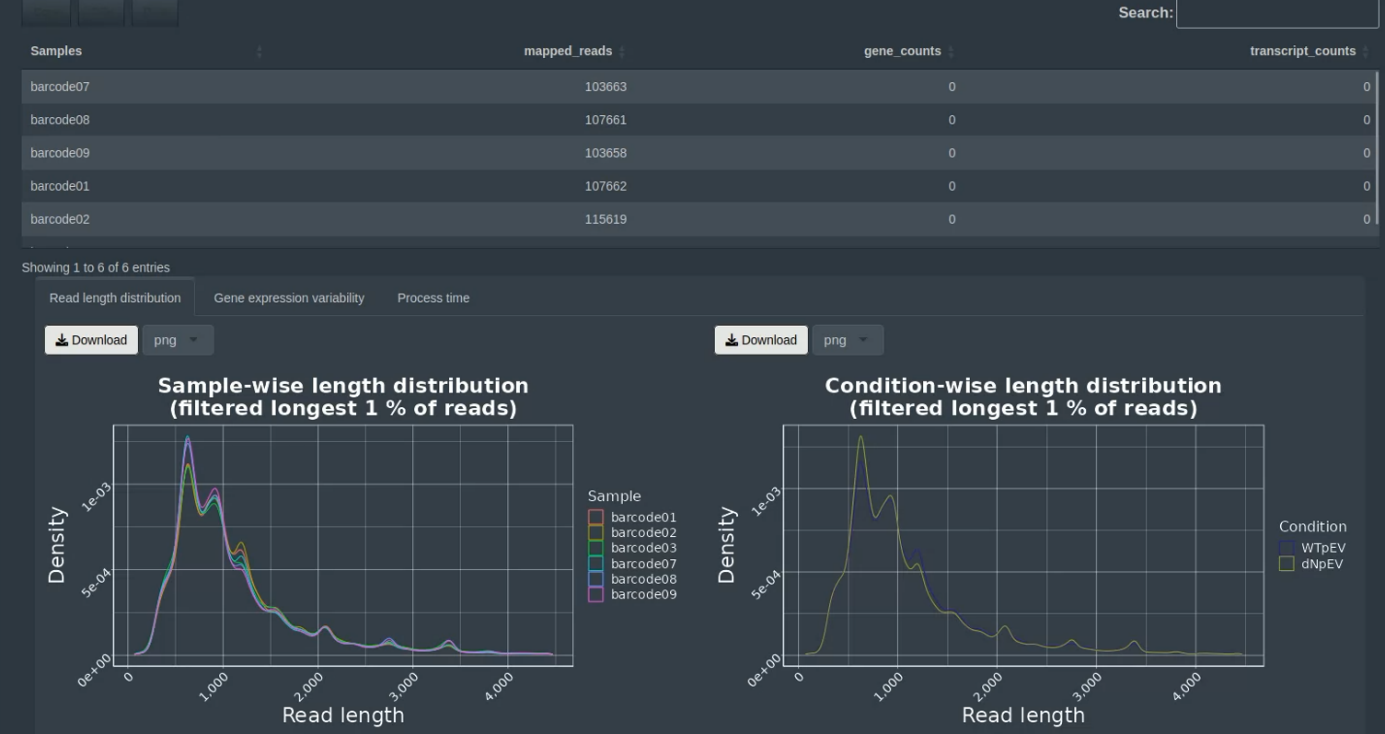
***Run Overview***- In “Run Overview”, users can check general information about the analyzed samples/conditions.
   - At the top of the page, users can see a table with barcoded samples, mapped reads, gene counts and transcripts. The table can be copied or exported as *csv* or *txt* files.


   - In the “Read length distribution” tab, users can have an overview of the read length overview per barcoded samples and conditions.
   - Figures can be exported as png figure by clicking the “download” button.


   - In the “Gene expression variability” tab, users can have an overview number of genes detected and changes in gene composition plot, per sample and per condition
   - Figures can be exported as png figure by clicking the “download” button.

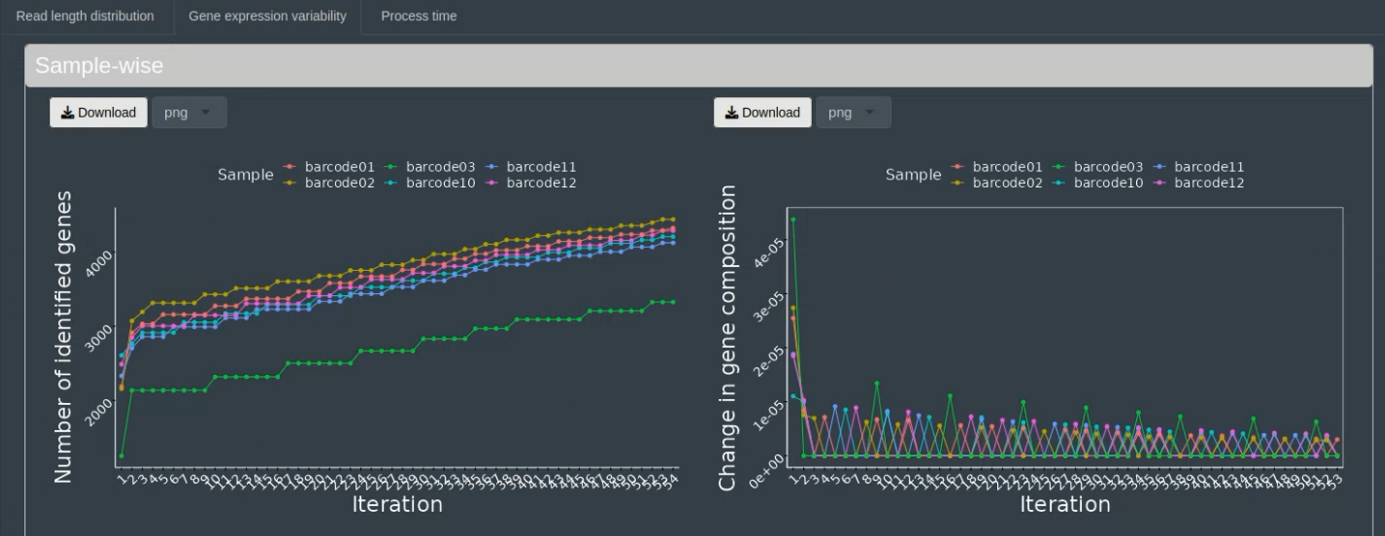


   - In the “process time” tab, users can have an overview of each tool’s processing times per iteration.
   - Figure can be exported as png figure by clicking the “download” button.

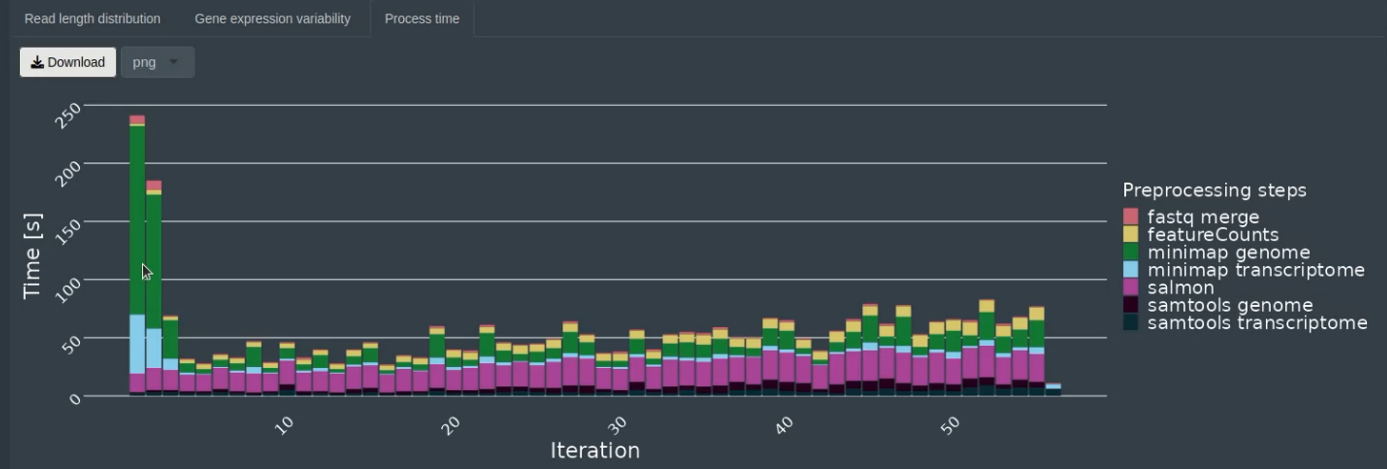

3.
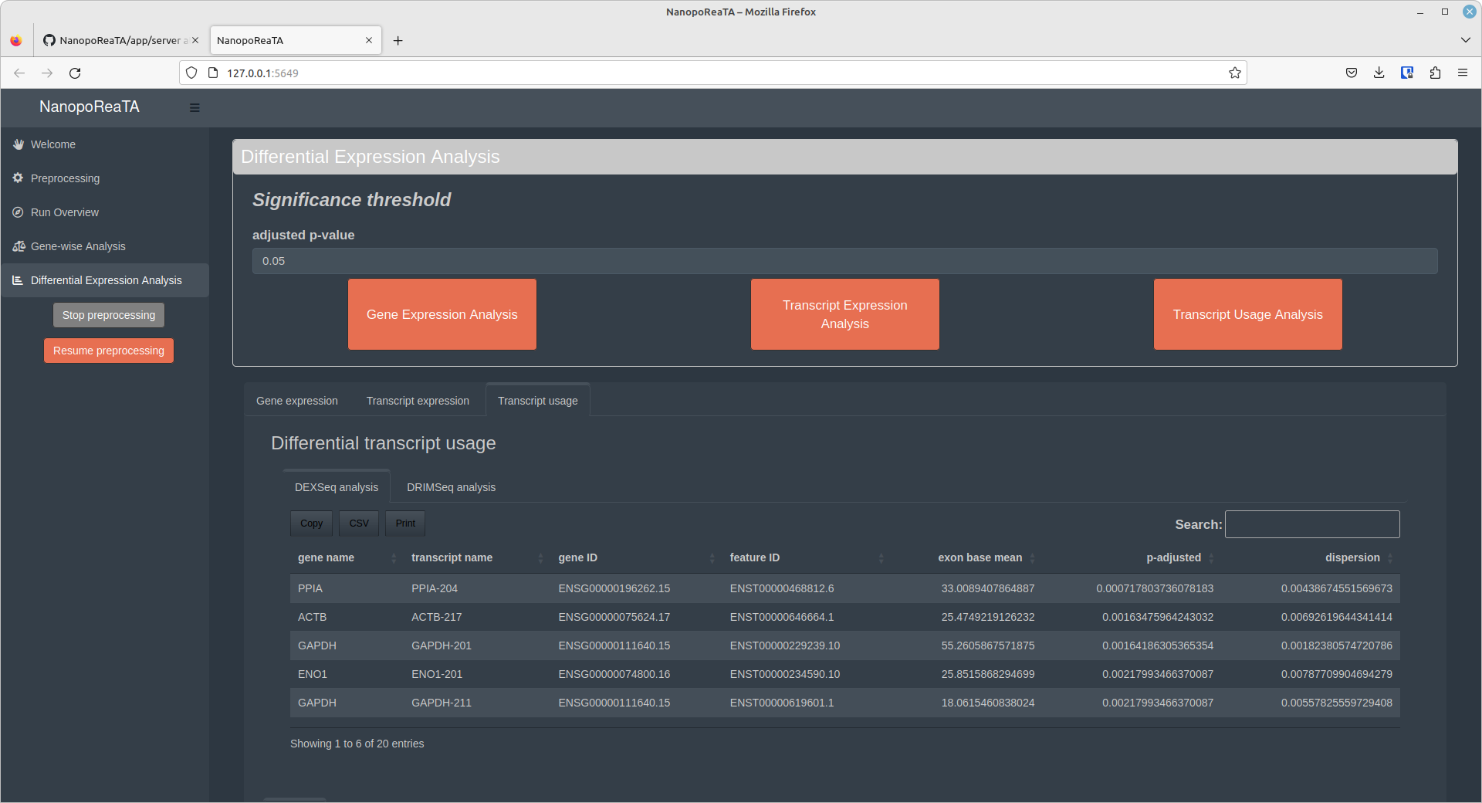
***Differential expression analysis***- In the "differential expression analysis" tab, users can choose the type of analysis they wish to perform, including "Gene expression analysis," "Transcript expression analysis," and "Transcript usage analysis".


   - Before activating any of the tools, it is important to follow these steps:

***Step 1*:** Press the "Stop preprocessing" button.

***Step 2*:** The button will turn grey, meaning the preprocessing of the sequenced files has stopped.

***Step 3*:** You can observe the last analysis steps being performed in the "process overview" tab.

***Step 4*:** Once the last analysis tool finalizes its task, it will be displayed in the "process overview" tab, and the "Resume preprocessing" button will become available.
***At this point, the activation of the "Differential Expression Analysis" tools is possible.***

*
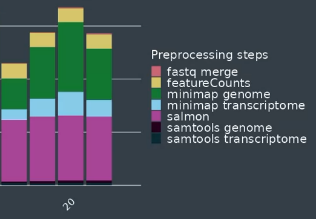
*
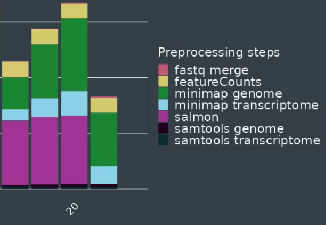

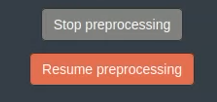

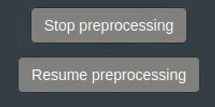

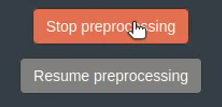
**Step 5:** To resume preprocessing additional files, press the "Resume preprocessing" button.

***Step 1***

***Step 2***

***Step 3***

***Step 5***

***Step 4***


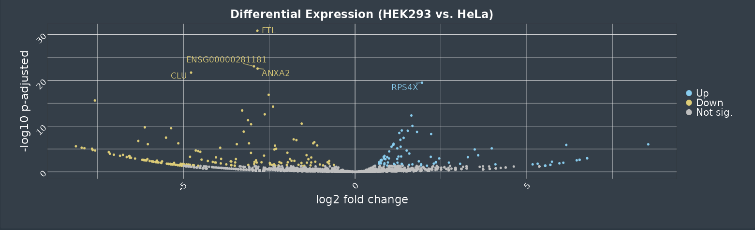

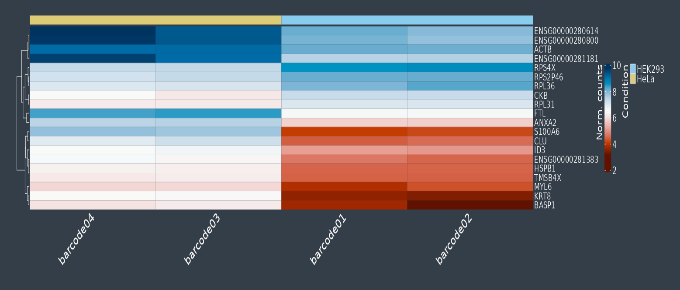

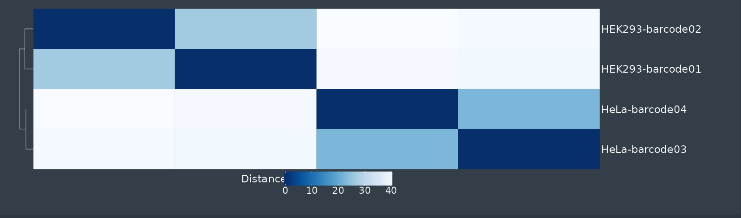

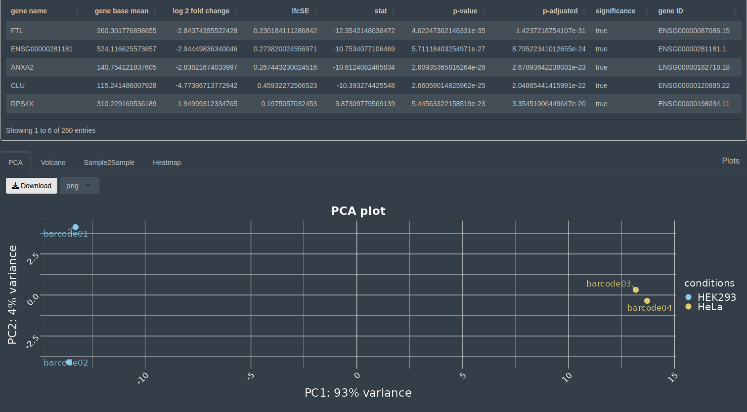

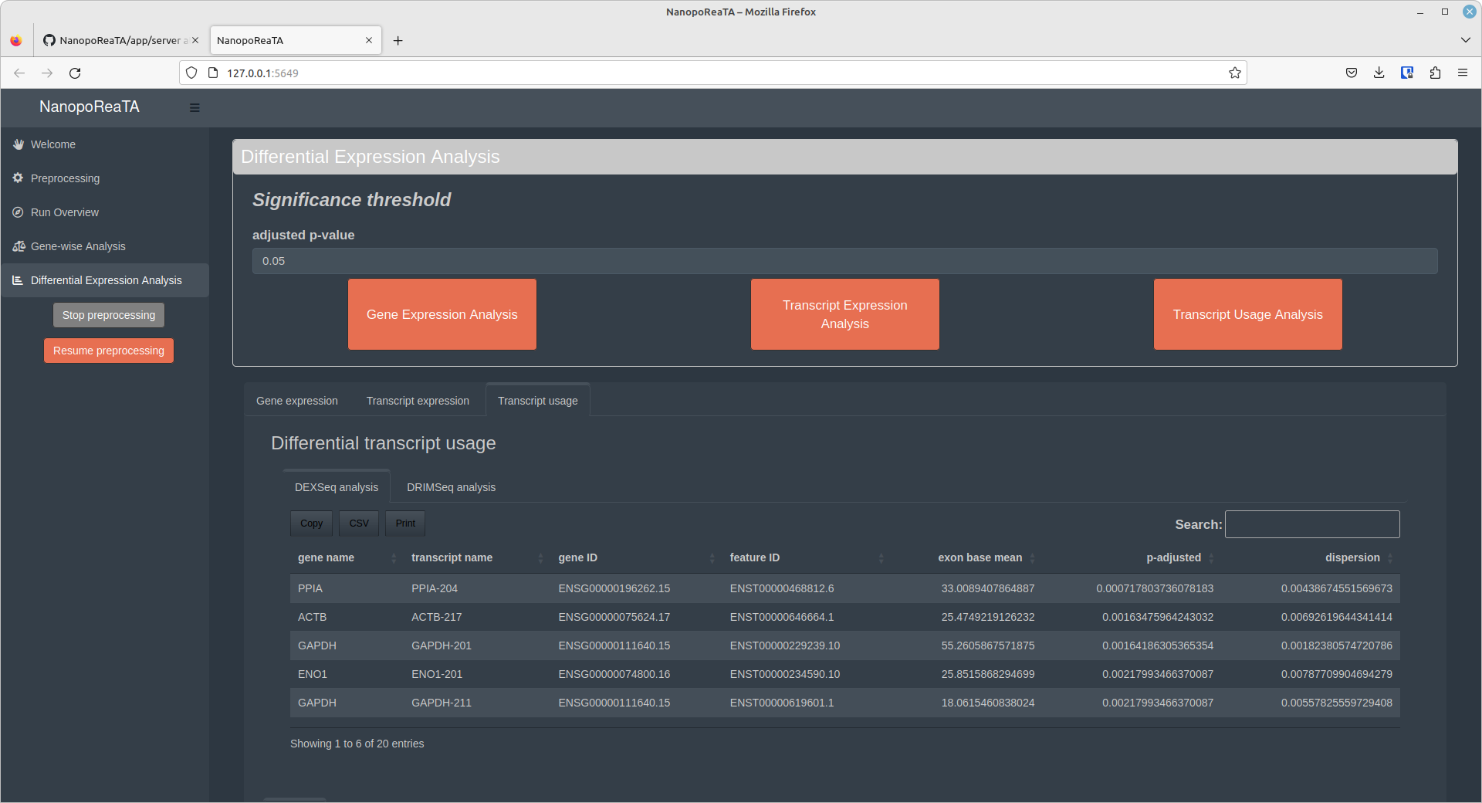

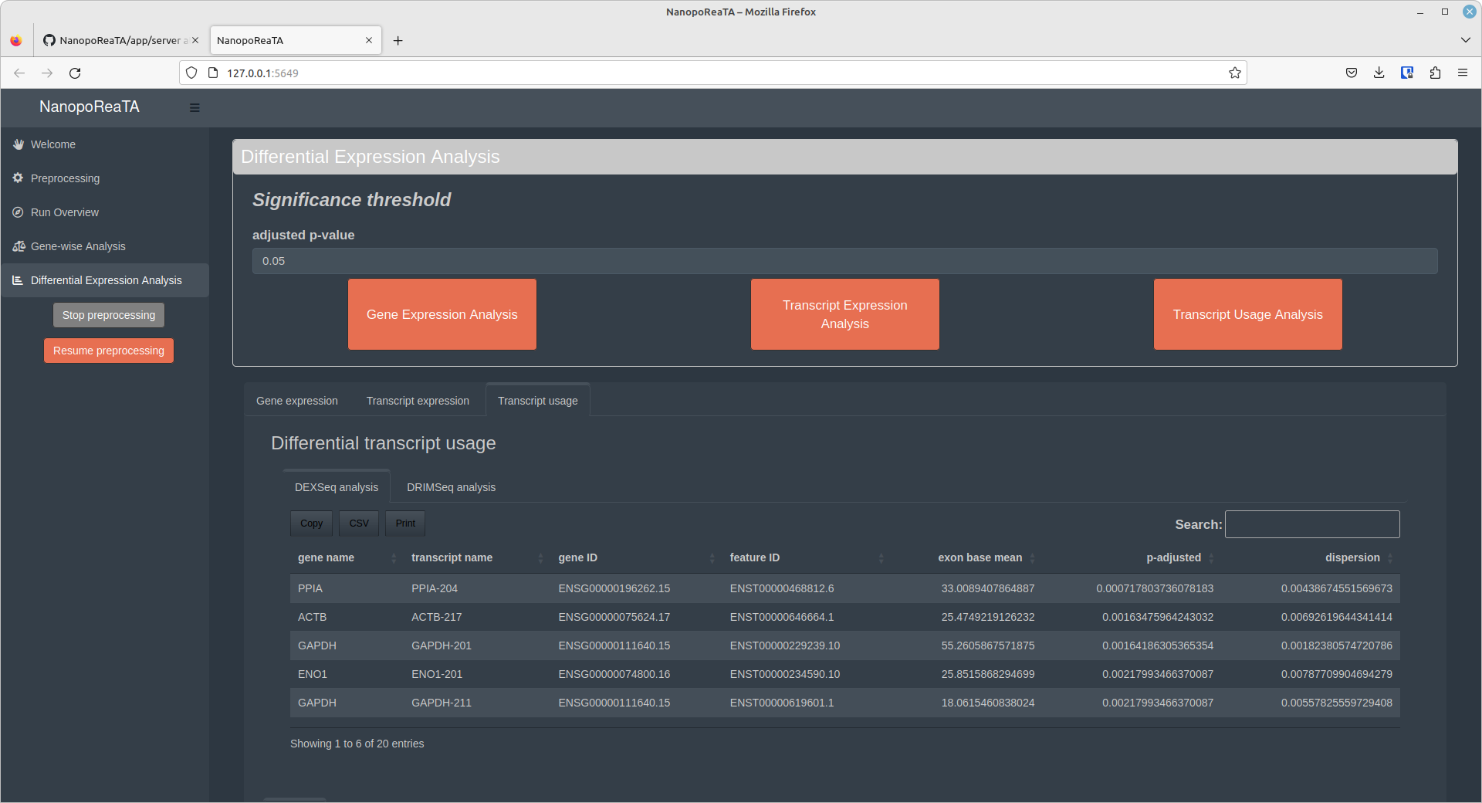
- Once one of the analysis tools is activated, users can check several analysis outputs provided by the tool shown in the “Gene expression”, “Transcript expression” and “Transcript usage” tabs.
- All tools will provide a table with differentially expressed genes/transcript and differentially used transcripts identified between the two conditions. The table can be copied or exported as *csv* or *txt* files.

- Gene expression and transcript expression analyses will provide tabs to visualize: PCA analysis, volcano plot (DGE), Sample-2-Sample plot and heatmap of the top 20 differentially expressed genes based on p-adjusted.
- All figures can be exported as png figure by clicking the “download” button.


- For differential transcript usage, users can select “general” or “gene specific” tabs.
- In “General” – users can visualize differentially used transcript in a volcano plot, which can be exported as png figure by clicking the “download” button.

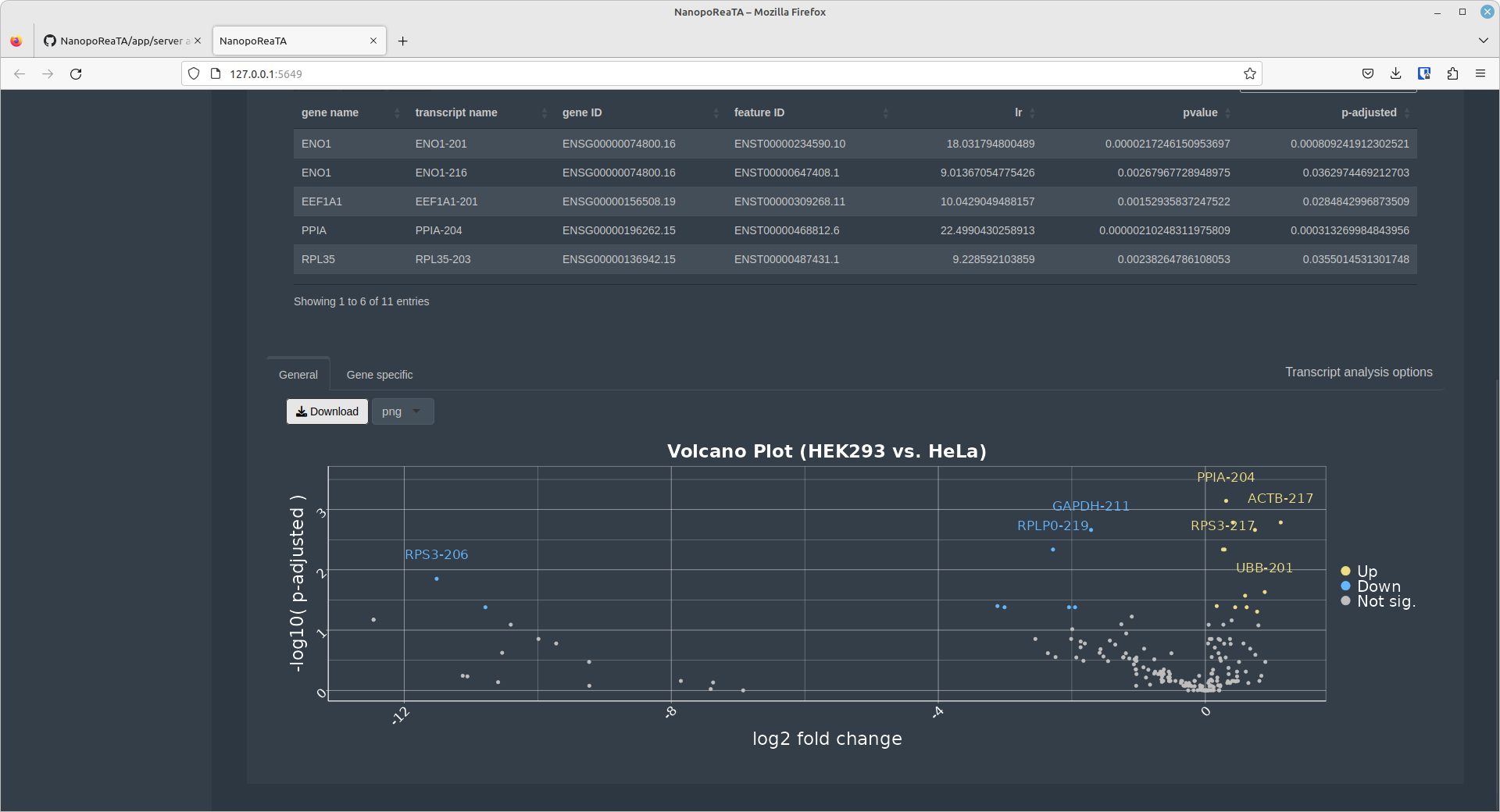


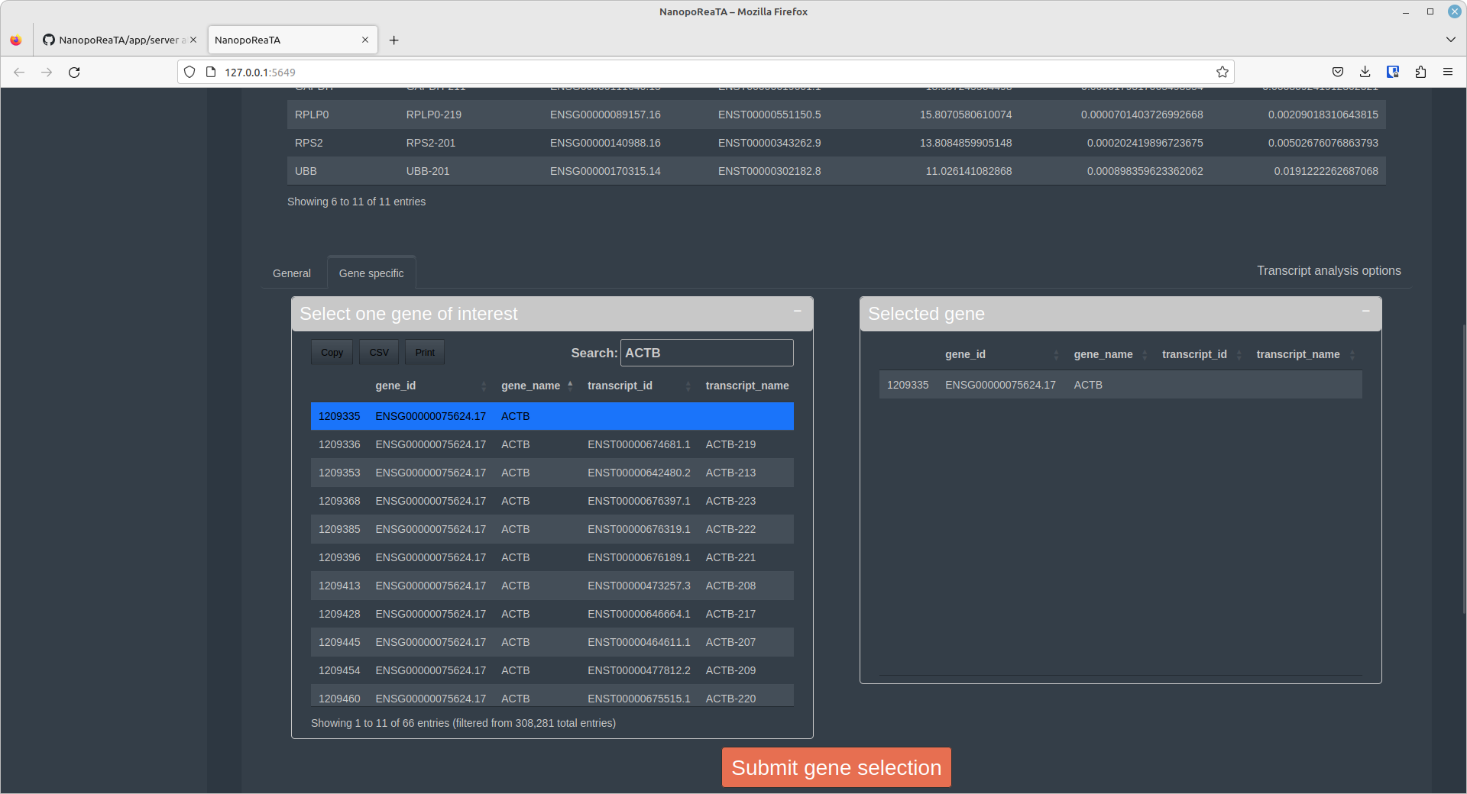


- In “Gene specific” – users can select a specific gene of interest and press “Submit gene selection”.

- After submission, a boxplot will appear, displaying the abundance of transcripts within a gene of interest for each condition, based on DRIMSeq's output. The figure can be exported as png figure by clicking the “download” button.


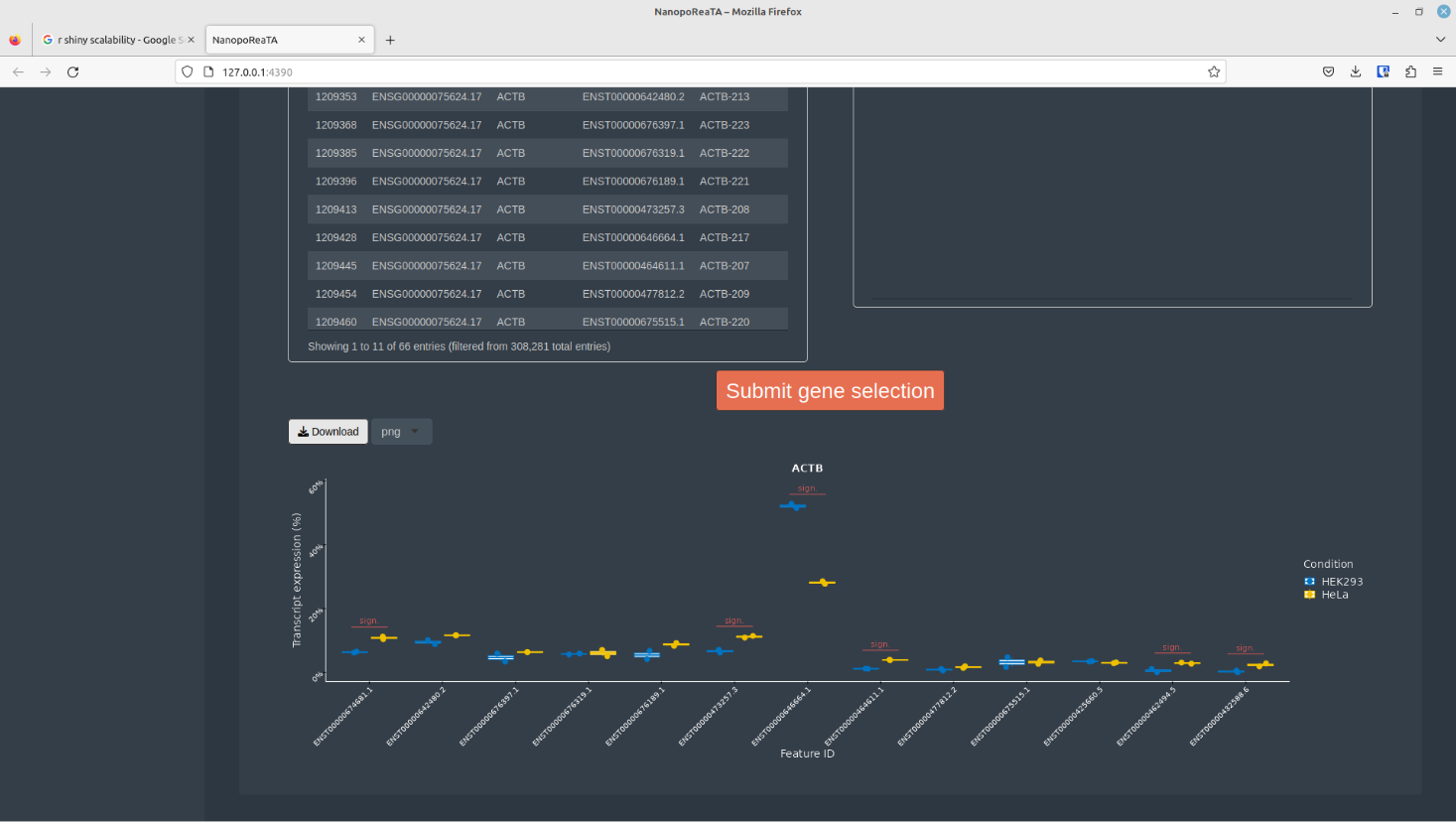


1. ***Gene-wise analysis***- In the "*Gene-wise analysis*" tab, users can choose between “Gene Counts” and “Gene Body Coverage” tabs.
   - In the "Gene Counts" - users can select multiple genes of interest by searching for the gene in the table and clicking on it. The selected gene will then be transferred to the "Selected genes" table, where users can click "Submit Genes" to proceed.


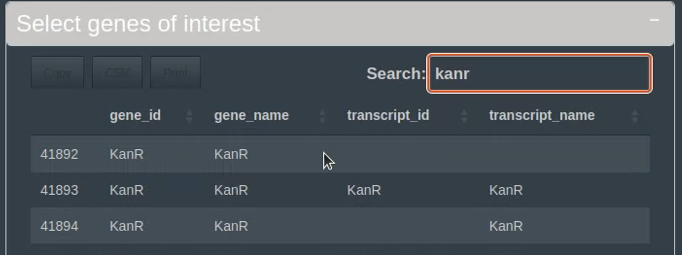


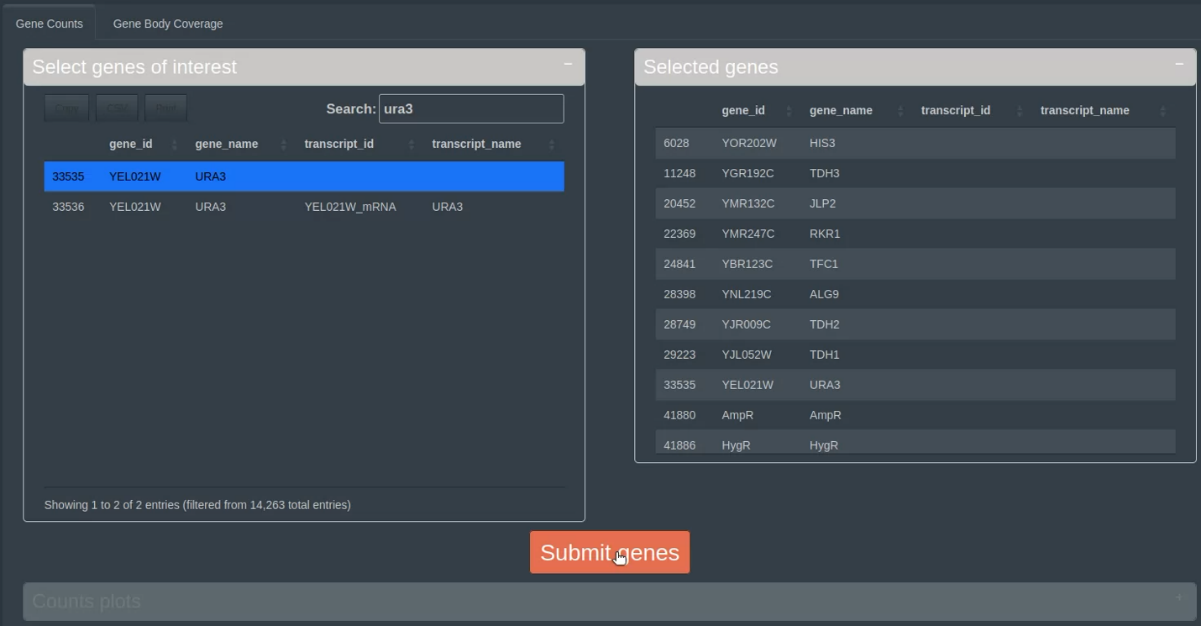


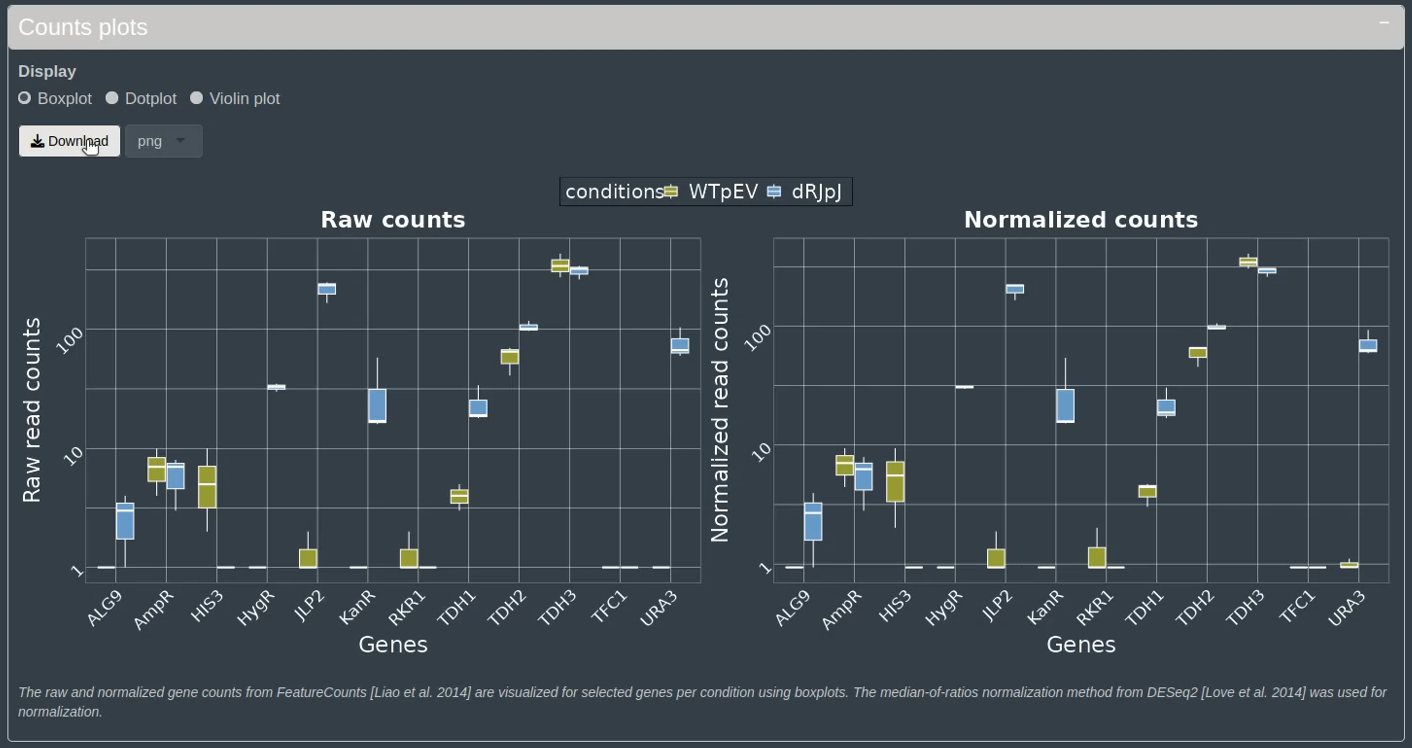
- a figure will appear showing a boxplot/dotplot/violin plot of the raw and normalized read counts per condition. The figures can be exported as png figure by clicking the “download” button.


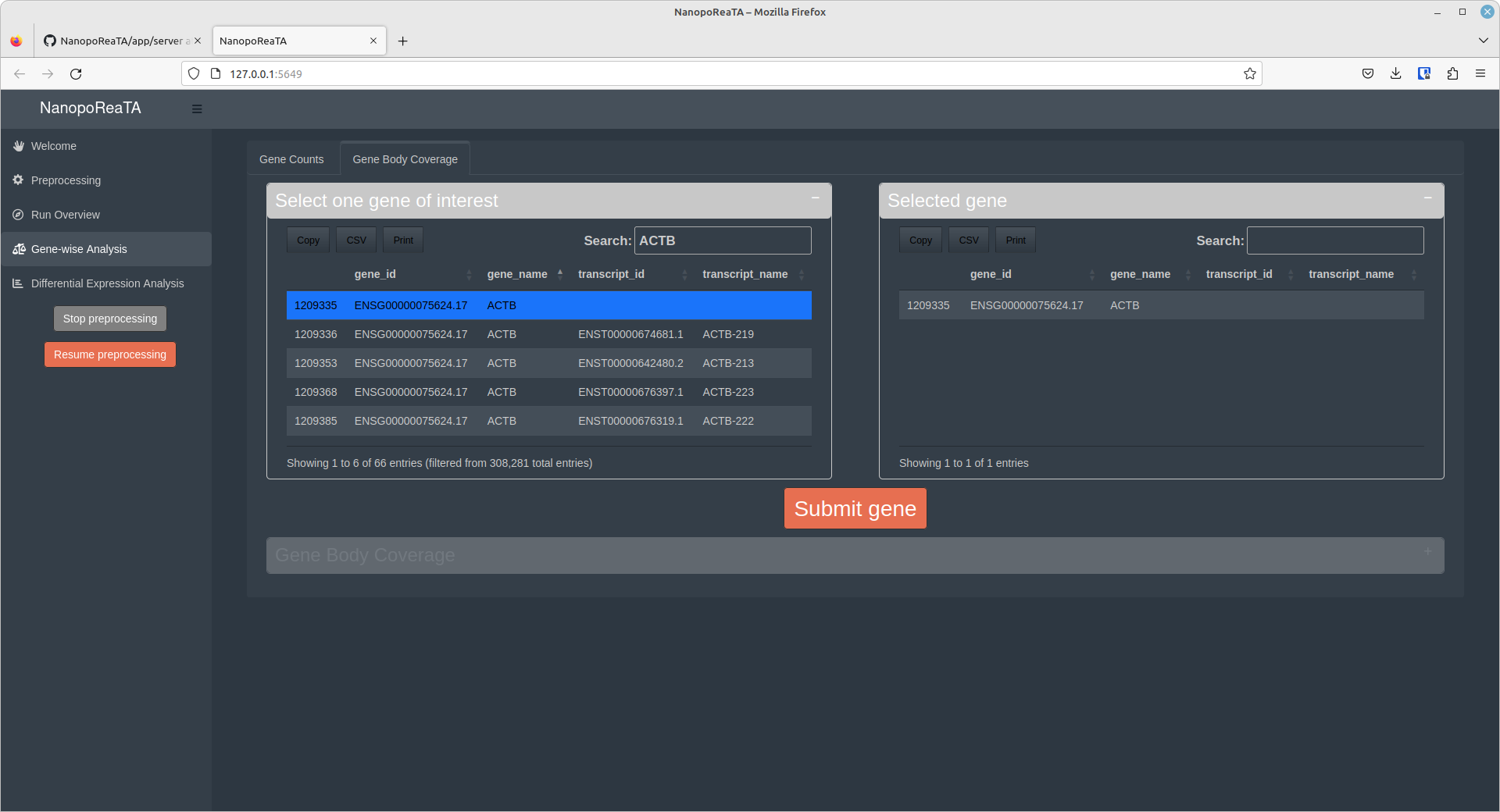
- In the “Gene Body Coverage”, users can select a specific gene of interest and press “Submit gene selection”.
- A figure will display, illustrating the percentage of coverage for exon percentiles for individual samples on the left side, and for the selected conditions on the right side. The figures can be exported as png figure by clicking the “download” button.


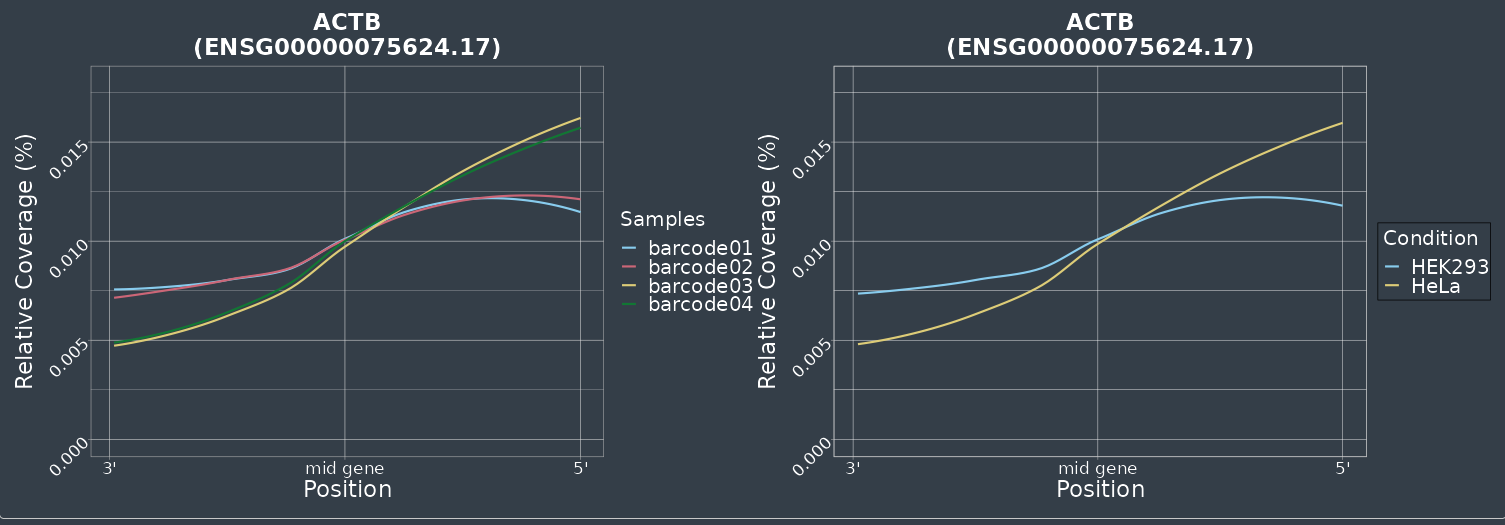

Supplement: Supplementary file 1. [file elife-98768-supp1.docx]
